# A near telomere-to-telomere genome assembly of *Isatis indigotica* L. facilitates phylogenomic studies in tribe Brassiceae

**DOI:** 10.1093/hr/uhag162

**Published:** 2026-05-13

**Authors:** Xiaohan Zhang, Yu Wang, Pingan Yu, Taihua Yang, Jing Wang, Zai yun Li, Xianhong Ge

**Affiliations:** National Key Laboratory of Crop Genetic Improvement, National Research Center of Rapeseed Engineering and Technology, Huazhong Agricultural University, Wuhan 430070, China; National Key Laboratory of Crop Genetic Improvement, National Research Center of Rapeseed Engineering and Technology, Huazhong Agricultural University, Wuhan 430070, China; National Key Laboratory of Crop Genetic Improvement, National Research Center of Rapeseed Engineering and Technology, Huazhong Agricultural University, Wuhan 430070, China; National Key Laboratory of Crop Genetic Improvement, National Research Center of Rapeseed Engineering and Technology, Huazhong Agricultural University, Wuhan 430070, China; National Key Laboratory of Crop Genetic Improvement, National Research Center of Rapeseed Engineering and Technology, Huazhong Agricultural University, Wuhan 430070, China; National Key Laboratory of Crop Genetic Improvement, National Research Center of Rapeseed Engineering and Technology, Huazhong Agricultural University, Wuhan 430070, China; National Key Laboratory of Crop Genetic Improvement, National Research Center of Rapeseed Engineering and Technology, Huazhong Agricultural University, Wuhan 430070, China

Dear Editor,

*Isatis indigotica* L. (2*n* = 14), a member of the tribe Isatideae within the *Brassicaceae* family, belongs to supertribe Brassicodae [1]. In this study, we present a near telomere-to-telomere (T2T) genome assembly of *I. indigotica* and develop specific markers for the precise identification of its centromeres. Both the roots and leaves of *I. indigotica* are used in traditional East Asian medicine. From phylogenomic perspective, the tribe Isatideae is an outgroup clade to the monophyletic tribes Brassiceae and Orychophragmeae [1]. *I. indigotica* possesses a typical translocation Proto-Calepineae Karyotype (tPCK), representing the ancestral karyotype prior to the whole-genome triplication event in the Brassiceae [2–4]. Our T2T genome assembly of *I. indigotica* will not only provide a comprehensive genomic resource for elucidating the biosynthesis and regulation of its medicinally active compounds, but also lay a foundation for tracing the evolutionary history of the Brassiceae genomes.

To construct a T2T genome for *I. indigotica*, we generated 33.59 Gb PacBio HiFi (~113.92× coverage). Four independent contig-level draft assemblies were produced by Hifiasm (v0.25.0), Canu (v2.2), Verkko (v1.13), and LJA (v2.2) software. Following a comprehensive comparative evaluation, the draft assembly from Hifiasm was selected as the backbone for subsequent refinement. This draft genome was 287.40 Mb in size, with a contig N50 of 33.61 Mb. Utilizing Hi-C data, 277.57 Mb of the sequence were successfully anchored onto seven pseudochromosomes, leaving only eight initial gaps. Six of these gaps were subsequently closed by integrating and crossvalidating sequence information from the other three draft assemblies, resulting in only two gaps located at 10.44 and 11.28 Mb on chromosome Iin4. The genome completeness assessed by The Benchmarking Universal Single-Copy Orthologs (BUSCO) reached 99.10%.

To ensure consistency, we have renamed the chromosomes from the original designations 0–6 to Iin1-Iin7, consistent with the tPCK karyotype [3]. Genome annotation was performed by a combination of *ab initio* prediction, homologous protein annotation, and transcriptome evidence, resulting in 29 136 annotated protein-coding genes. The assembly includes 44.27% repetitive sequences, and BUSCO assessment demonstrated a high completeness at 97.0%. This represents a significant improvement over the previous annotation completeness of 95.7% for this species. Specifically, there has been an increase of complete and single-copy genes from 67.5% to 94.0%, while complete and duplicated genes have decreased from 28.2% to 3.0%. These results indicate significant enhancements in both assembly and annotation quality (Fig. 1A–C).

**Figure 1:**
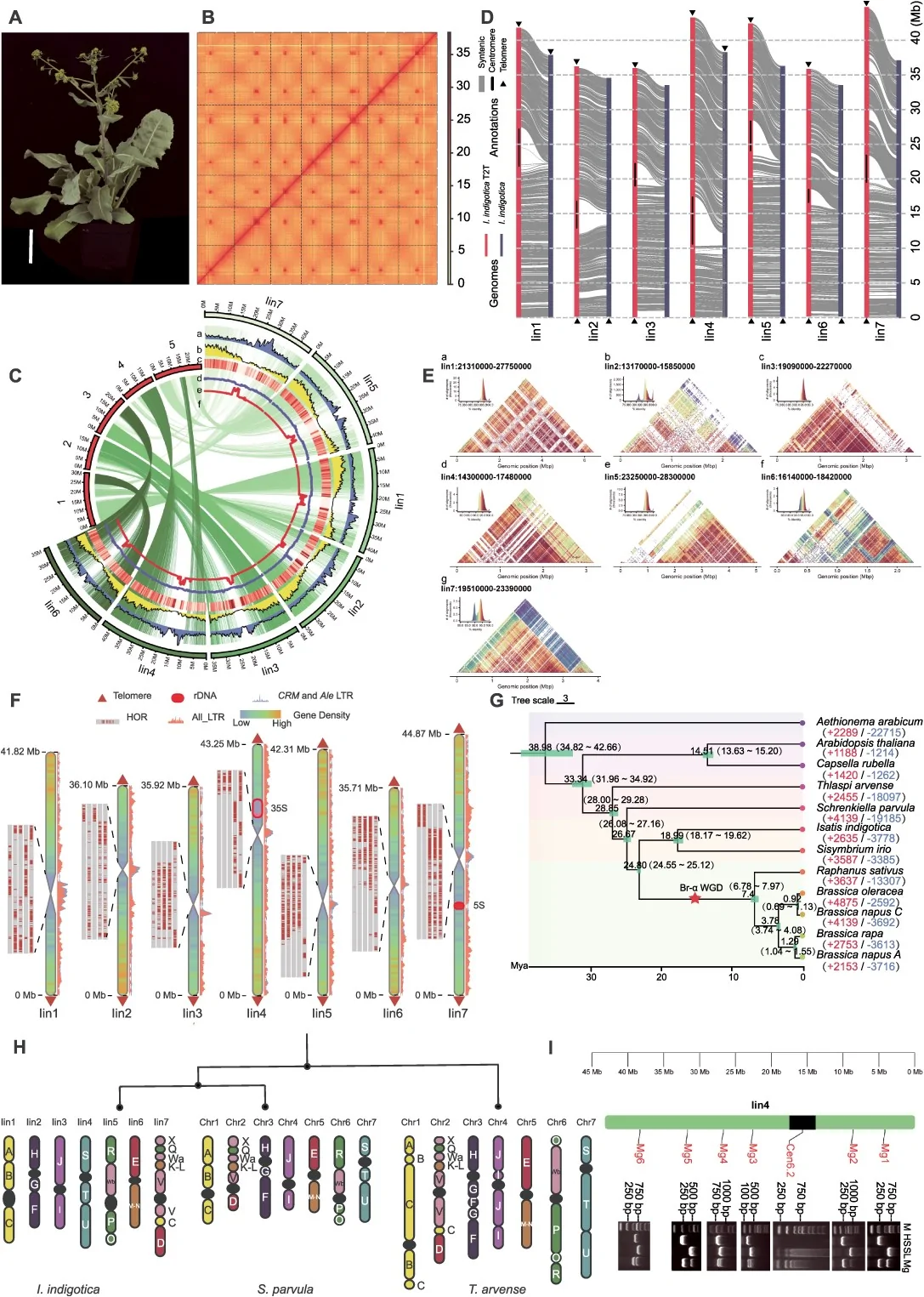
Overview of T2T genome and comparative genome analysis of *Isatis indigotica.* (A) Morphology of *I. indigotica.* Scale: 15 cm*.* (B) Whole Genome Hi-C map of *I. indigotica.* (C) Genomic feature of *I. indigotica*. (a. GC content. b. Gene density. c. SNP density. d. Terminal Inverted Repeat (TIR) density. e. Long Terminal Repeat (LTR) density. f. Genome collinearity between I. *indigotica* and *A. thaliana*). (D) Genomic collinearity between two *I. indigotica* genomes. (E) Overview of all centromeric regions in the *I. indigotica* assembly. StainedGlass heatmaps show the sequence identity between 5-kb bins within whole centromeric regions. (F) Chromosome karyotype and centromere characteristics of *I. indigotica*. Telomeres are marked by triangles. Gene density is represented by chromosomal intensity gradients. Boxed chromosomal segments indicate the genomic distribution of ribosomal DNA (rDNA) arrays. Two tracks on the right side of the chromosomes represent the whole-genome LTR density and the distribution density of CRM and Ale LTRs. The 5 left bar plots per chromosome represent the relative abundance distribution of the five most prevalent higher-order repeat (HOR) variants within centromere. (G) Phylogenetic tree of 12 species. Numbers at nodes indicate divergence times, and the numbers of expanded and contracted genes are shown below each species. (H) Karyotype of *I. indigotica*, *Schrenkiella parvula* and *Thlaspi arvense*. The sizes of genome blocks were kept consistent with their actual genomic dimensions. (I) Electropherograms for distinguishing *B. napus*- *I. indigotica* additional line using centromere markers and co-dominant markers on chromosome arms.

Compared with previous genome assemblies, the T2T genome exhibited improved collinearity, accompanied by an increased pseudochromosome anchoring rate (Fig. 1D) [3]. We predicted the centromeric positions of *I. indigotica* by analyzing HiFi and Hi-C data using the RepCent software. The results showed that centromeres were identified on all chromosomes, with sizes ranging from 2.44 to 6.28 Mb (Fig. 1E). Furthermore, diverse higher order repeat arrays were identified across all seven chromosomes using HiCAT, with unique monomer amplifications being abundant and highly variable in size within the centromeres (Fig. 1F). A 148-bp centromeric satellite repeat was identified on all chromosomes. Our results also indicate that the satellite repeats in the centromeres of *I. indigotica* are not closely and consecutively arranged, and the centromere structures of the seven chromosomes have been invaded by long terminal repeats (LTRs) to varying degrees. In light of this result, we performed a detailed analysis of the distribution and insertion time of LTRs across the *I. indigotica* genome. Among the 139.35 Mb of annotated repetitive sequences, *Copia* and *Gypsy* retrotransposons (RTs) accounted for 9.95% and 20.87%, respectively. Analysis of the insertion times for 3058 full-length RTs (1683 *Copia* and 1151 *Gypsy*) revealed an average insertion time of approximately 0.087 million years ago (Mya). Notably, 331 full-length LTR RTs located within centromeric regions showed a significantly younger average insertion time of ~0.016 Mya, compared to ~0.10 Mya for LTRs in other genomic regions (*P* < 0.0001). We identified 373 *CRM* (*Gypsy*) elements, with 183 located in centromeres and 86 intact *Ale* (*Copia*) elements, which represent the predominant LTR type within centromere regions. These results indicate that nested LTR insertions, preferentially targeting centromeric regions, disrupting the structure of centromeric satellite repeats and likely drive rapid centromere evolution, consistent with previous findings in *Brassica rapa* [5]. Thirteen complete telomeric arrays were identified by quartet (v1.1.6), with only the left telomere of Iin1 missing. The plant-specific telomeric repeat (TTTAGGG) was found with repeat numbers ranging from 278 to 482 (Fig. 1D and E).

A species divergence time tree was constructed using 2492 single-copy genes from 12 species (Fig. 1F). The results indicate that *I. indigotica* diverged from *Brassica* species approximately 24.98 Mya and experienced its most recent whole-genome duplication (WGD) event around 33.34 Mya, consistent with the At-α WGD shared by all *Brassicaceae* species. Compared to *Sisymbrium irio*, *I. indigotica* exhibited expansions in 2635 gene families and contractions in 3778 gene families. Gene family analysis across 11 species enabled the construction of a high-confidence set of 12 736 orthologous genes. Positively selected genes (PSGs) refer to advantageous genes that have been preserved through natural selection during the process of evolution. Rapidly evolving genes (REGs) refer to genes that undergo rapid mutations during the evolutionary process, often associated with characteristics during species formation, and may be key factors leading to the antiviral activity of *I. indigotica*. Using the branch-site and branch models in PAML4.9j, we assessed differences in adaptive evolution between *I. indigotica* and other plants. This analysis identified 626 PSGs and 1525 REGs. Kyoto Encyclopedia of genes and genomes (KEGG) enrichment analysis revealed that these genes are primarily involved in crucial biological pathways such as plant hormone signal transduction, pentose and glucuronate interconversions, and ubiquinone and terpenoid-quinone biosynthesis. These genes may be closely associated with the formation of medicinally active secondary metabolites in the leaves and roots of *I. indigotica*.

To further characterize the karyotype of *I. indigotica*, we performed a comparative analysis using 22 ancestral genome blocks defined for the ancestral *Brassicaceae* karyotype (Fig. 1H). Although *I. indigotica* is characterized as having a standard tPCK [3], our assembly reveals several structural deviations, particularly on chromosome Iin7, distinguishing it from other tPCK-type species such as *Schrenkiella parvula* and *Thlaspi arvense*. In chromosome Iin7, an extra C block (AT1G54450-AT1G55040) was detected between the D and V blocks, forming a new block association of D-C-V-K-L-Wa-Q-X. Furthermore, the V Block exhibits a spanning pattern across the centromere (Fig. 1H). This result reveals a clearer tPCK, which will provide a basis for the refined delineation of karyotype block boundaries in *Brassicaceae* chromosomes and lay a foundation for future studies on karyotype evolution in *Brassica* species. Based on the characterization of satellite repeats in centromeric regions and collinearity analysis between *B. napus* and *I. indigotica*, one centromere-specific and six co-dominant molecular markers for chromosome Iin4 were developed. These markers identify the whole Iin4 chromosome and thus effectively distinguish *B. napus* and *B. napus*–*I. indigotica* Iin4 addition lines (Fig. 1I).

In conclusion, we have assembled a T2T genome for *I. indigotica*, which is nearly complete except for the missing left telomere of Iin1 and two gaps on the arm of chromosome Iin4. This represents a significant advancement in quality, particularly for the assembly of centromeric regions and the comprehensive reannotation of the genome. This will undoubtedly greatly facilitate the precise capture of chromosome structural variations during the genome triplication of Brassiceae species, providing more clues for understanding the adaptive evolution of chromosome structural variations. Phylogenomic identification of PSGs and REGs will also facilitate the identification of genes as well as related medicinally active metabolites in this medicinal plant.

## Data Availability

All the raw sequencing data, assembly and annotation results of *Isatis indigotica* T2T genome generated during the current study are available in the China National Center for Bioinformation BioProject under accession number PRJCA053928.
